# Targeting male mosquito swarms to control malaria vector density

**DOI:** 10.1371/journal.pone.0173273

**Published:** 2017-03-09

**Authors:** Simon Peguedwinde Sawadogo, Abdoulaye Niang, Etienne Bilgo, Azize Millogo, Hamidou Maïga, Roch K. Dabire, Frederic Tripet, Abdoulaye Diabaté

**Affiliations:** 1 Department of Medical Biology and Public Health, Institut de Recherche en Science de la Santé (IRSS), Bobo-Dioulasso, Burkina Faso; 2 Deparment of Population Sciences, Institut des Sciences des Sociétés (INSS), Ouagadougou, Burkina Faso; 3 Centre for Applied Entomology and Parasitology, School of Life Sciences, Keele University, Staffordshire, United Kingdom; University of Crete, GREECE

## Abstract

Malaria control programs are being jeopardized by the spread of insecticide resistance in mosquito vector populations. It has been estimated that the spread of resistance could lead to an additional 120000 deaths per year, and interfere with the prospects for sustained control or the feasibility of achieving malaria elimination. Another complication for the development of resistance management strategies is that, in addition to insecticide resistance, mosquito behavior evolves in a manner that diminishes the impact of LLINs and IRS. Mosquitoes may circumvent LLIN and IRS control through preferential feeding and resting outside human houses and/or being active earlier in the evening before people go to sleep. Recent developments in our understanding of mosquito swarming suggest that new tools targeting mosquito swarms can be designed to cut down the high reproductive rate of malaria vectors. Targeting swarms of major malaria vectors may provide an effective control method to counteract behavioral resistance developed by mosquitoes. Here, we evaluated the impact of systematic spraying of swarms of *Anopheles gambiae* s.l. using a mixed carbamate and pyrethroid aerosol. The impact of this intervention on vector density, female insemination rates and the age structure of males was measured. We showed that the resulting mass killing of swarming males and some mate-seeking females resulted in a dramatic 80% decrease in population size compared to a control population. A significant decrease in female insemination rate and a significant shift in the age structure of the male population towards younger males incapable of mating were observed. This paradigm-shift study therefore demonstrates that targeting primarily males rather than females, can have a drastic impact on mosquito population.

## Background

Malaria, a preventable disease, is still one of the most widespread causes of morbidity and mortality in developing countries, especially in sub Saharan Africa [1]. Successive WHO World Malaria reports clearly indicate a significant decrease in its incidence in the past 15 years, confirming that millions of lives have been saved by increased investments in health made over the same period [2,3,4,5,6,7,8,9]. Globally, the declining number of malaria cases is estimated at 18%, from 262 million in 2000 to 214 million in 2015. Meanwhile the associated mortality has decreased by 48%, from 839,000 deaths in 2000 to 438 000 in 2015 (WHO, 2015). Most malaria cases and related deaths occur in the WHO African region (88%), far ahead of the Southeast Asia region [1]. Vector control implemented mainly through Indoor Residual Spraying (IRS) campaigns and the distribution of Long-lasting insecticidal nets (LLIN) has played an important role in decreasing the incidence of the disease [10] and is typically used in combination with drug treatments.

LLINs and IRS effectively kill malaria vectors that bite people indoors and rest inside human dwellings at night. However, in the last few years, malaria vectors biting outdoors and/or in the evening and early morning, when people are not protected by these conventional tools, has been recorded in several studies [11,12,13]. By adapting to seeking hosts outdoors, malaria vectors avoid LLINs or IRS and picking up a lethal dose of insecticide. The increasing importance of outdoor-biting by malaria vectors indicates a major limitation to current vector control based primarily on LLINs and IRS, both of which are indoor interventions [14]. Moreover, other than resting and blood-feeding, there are numerous other mosquito life cycle stages that predominantly occur outdoors and are not amenable to LLINS and IRS, thus requiring new outdoor measures [15,16,17]. Examples of these outdoor activities include oviposition site seeking, foraging for sugar meals, outdoor resting, and swarming, which is believed to take place solely for mating purposes [15,16,17].

Recent developments on our understanding of mosquito swarming give hopes that new tools targeting mosquito swarms can be designed to cut down the high reproductive rate of malaria vectors. Mating is a vulnerable step in the *An*. *gambiae* s.l life cycle, as females of this species only mate once in their lives [12,13]. Mating occurs in swarms formed by males around dusk [14], females approach a swarm, acquire a mate, and leave in copula. We showed in previous studies that swarms of *Anopheles gambiae* s.l. [15,16,17] use distinctive landmarks to gather and mate. They systematically use the same sites over time. The predictability of the sites makes them an easy target to control mosquito reproduction rate by killing not only large number of males but also visiting females. Targeting swarms of major malaria vector could be a good alternative control method in response to behavioral resistance developed by these mosquitoes. In addition a recent study in Bama area reported a low level of resistance to carbamates [18]. In this study, we evaluated the impact of mass killing swarms of *Anopheles gambiae* s.l. using an aerosol KALTOX^®^, a mixture of carbamate (Propoxur) and a Pyrethrinoid (Alletrin, Tetrametrin and Permetrin), and estimated the impact of this intervention on vector density, female insemination rates and male age structure inferred from scoring of genitalia rotation.

## Materials and methods

### Study area

The study was carried out in the area of Bama (11°24’29”N; 04°24’37”W). It consists of a cluster of villages ~30 km north-west of Bobo-Dioulasso, in the valley of the Kou River, a region of extensive rice cultivation that was established during the 1970s. Seven villages, named VK1-7 and covering 7,200 ha were created as part of an irrigation development scheme. Each village lies at the edge of rice fields. The Kou River offers a permanent source of water for irrigation, enabling the growth of two crop seasons of rice per year (July-November and January-May). As a result the rice fields constitute a highly productive breeding habitat for mosquitoes for large portions of the year. More typical natural anopheline breeding sites (rain puddles and rain or ground-water filled depressions, such as tyre tracks) are also present. Both *An*. *coluzzii* and *An*. *gambiae* have been recorded at high densities during the rainy season (May-October), with typical biting rates for *An*. *coluzzii* of ~ 200 bites/person/night (Baldet et al 2003). Killing males in masse as well as mate seeking females by targeting swarms at sunset with bombs spray as way of controlling mosquito density was carried out in VK5 in September 2013. VK7, the closest village from VK5 with similar ecological setting was selected as a control village.

### Study procedure

**Fig 1:**
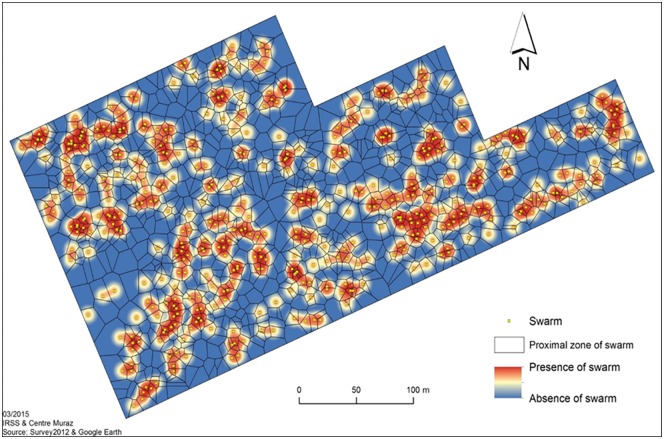
Map distribution of swarms in VK5 village.

The study was carried out over 1 month in September 2013. Prior to the intervention, a first round of baseline entomological data was collected in both villages to estimate mosquito density, female insemination rate and the age structure of males for 5 days. The estimate of mosquito density was done by pyrethrum spray catch. A total of 20 houses were randomly selected in each village. Pyrethrum spray catch was performed in these houses by knocking down on a white spreadsheet resting mosquito in the rooms with a bomb spray. Morphological identification of collected mosquitoes was done and only females belonging to *An*. *gambiae* s.l were counted. A subset of 240 females/village was sampled and their spermatheca dissected to look at their mating status. In addition, males were collected in 10 randomly selected inhabited houses by vacuum aspiration across each village to check on the male population age structure. A subset of 240 males was sampled from the pool and their genitalia observed [19]. Newly emerged males cannot mate. Sexual maturation is completed 24 hours or more after emergence and that requires a 180° rotation of the genitalia. The rate of genitalia rotation was used as a proxy to estimate the age structure of male population. In addition to this set of activities, an extensive search of swarms was done in the VK5 village. A total of about 300 swarms were spotted in the village and a map of the distribution of swarms was constructed (Fig 1).

**Fig 2:**
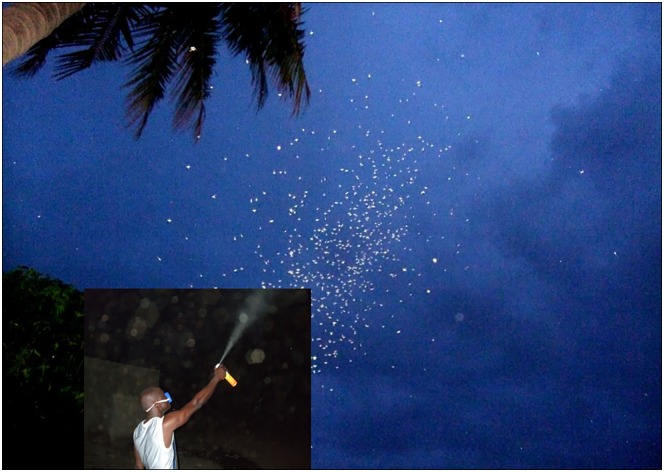
A volunteer spraying swarms with a bomb spray.

During the intervention phase, 20 volunteers recruited from the VK5 village, were trained to target swarms with bomb spray containing a mixture of pyrethroid and carbamate (Fig 2). Volunteers were shown the map of swarm distribution in the village and each of them was assigned 2–4 contiguous compounds and asked to target swarms with the bomb spray in these compounds. Every day at sunset and for 3 consecutive days, males and mate seeking females were massively killed in swarms by spray. Given the number of volunteers involved and the size of the village, sprayers were able to systematically target all mosquitoes present in swarms across the village already on the first day of the intervention. On the second and third days volunteers inspected scrupulously all swarm sites across the village and whenever a swarm was detected it was systematically sprayed to kill all mosquitoes. On subsequent days, the spraying was done every two days for a total of 6 days resulting in a total intervention period of 9 days. Following this, a second round of entomological data, similar to that collected during the pre-intervention period, was collected again in both villages to estimate mosquito density, female insemination rate and the age structure of males post-intervention. The amount of insecticide sprayed in the environment could not be measured in this study. However, given that the insecticide was specifically sprayed on swarms and the fact that swarms are found at discrete places prevent a blind spraying of insecticide in the environment.

### Data analyses

Data were entered and cross-checked in Windows Excel 2007. Statistical analyses were performed using R 2.12 with a significance level of 5%. Three main variables were analyzed: density of mosquitoes per house, female insemination rates, proportion of immature males. ANOVAs were conducted to compare the mosquito density between the intervention and the control villages, followed by Tukey’s pairwise comparisons tests. For insemination rates and the proportion of immature males a Kruskal-Wallis test was used, followed by Mann-Whitney pairwise comparisons test.

### Ethical considerations

Our study did not involve human patients. The full protocol of the study was submitted to the institutional ethics committee of l’Institut de Recherche en Sciences de la Sante for review and approval (inclure ici les references de l’autorisation). In accordance with the approval, visits were done in the study sites to present the project and to request the participation of villagers. During these visits the objectives, protocol and expected results were explained and discussed with the villagers, as well as the implications for the households willing to take part in this study. A written consent form was signed or marked with fingerprint by the head of the households before any activity could take place in his compound. Insecticides used in this study are approved for use by the Burkina Faso insecticide regulation authority. They are commonly used indoors and outdoors by villagers to kill mosquitoes and other insects.

## Results

*An*. *gambiae* s.l. swarms were geo-positioned using GPS coordinates through the whole village. In VK5 all identified swarms were sprayed to kill both males and mate seeking females visiting swarms.

### Impact of the intervention on the mosquito densities (Fig 3)

**Fig 3:**
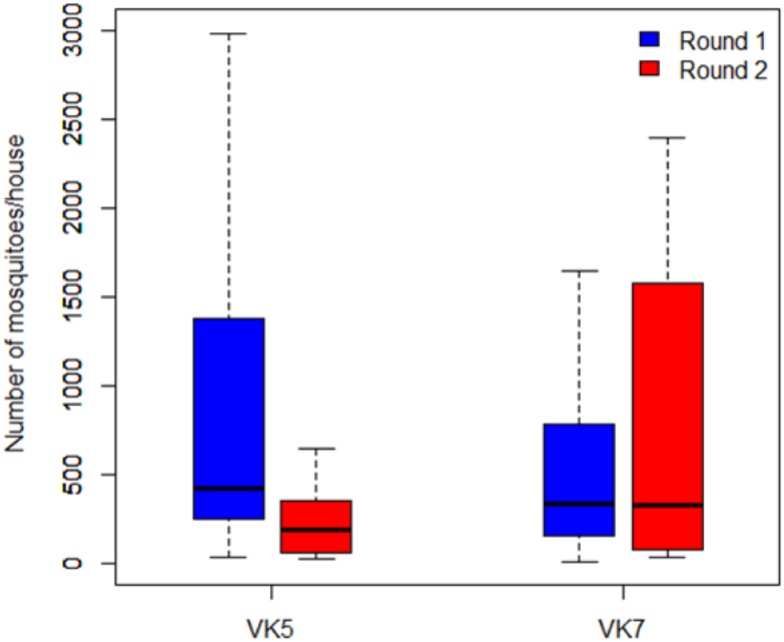
Mosquito densities in the villages of VK5 and VK7 pre-intervention (Round 1 in blue) and post-intervention (Round 2 in Red).

Overall, a total of 22,269 *An*. *gambiae* s.l. mosquitoes were collected in VK5 and 29,028 in VK7 in the human dwelling.

In VK5 the intervention village, the results show that the number of mosquitoes per house was significantly higher in Round 1, prior to the intervention, compared to Round 2 that followed the intervention (t = 3.16; df = 20.88; P = 0.0047). On average, mosquito density decreased from 882.85 mosquitoes/house in the Round 1 to 230.57 mosquitoes/house in Round 2. Therefore, a dramatic reduction of 73.88% in mosquito density was observed as a direct impact of the intervention. In contrast, in the control village VK7, no difference in mosquito density was found between the two rounds (t = -0.87; df = 32.35; P = 0.38).

### Impact of the intervention on the insemination rate in female mosquitoes (Fig 4)

**Fig 4:**
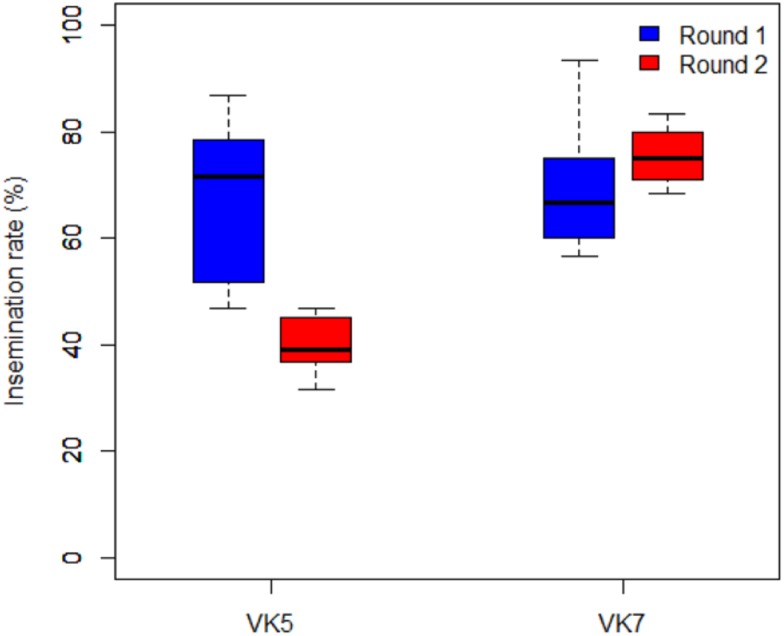
Insemination rates in the villages of VK5 and VK7 pre-intervention (Round 1 in blue) and post-intervention (Round 2 in Red).

In VK5, the insemination rate was significantly higher in Round 1 (67.08%) than in Round 2 (40%) (W = 63; P = 0.0012). In the control village VK7, average insemination rates did not vary significantly between the two rounds (W = 15.5; P = 0.1), and were high in Round 1 (69.17%) and in Round 2 (75.42%).

### Impact of the interventions on the age structure of males (Fig 5)

**Fig 5:**
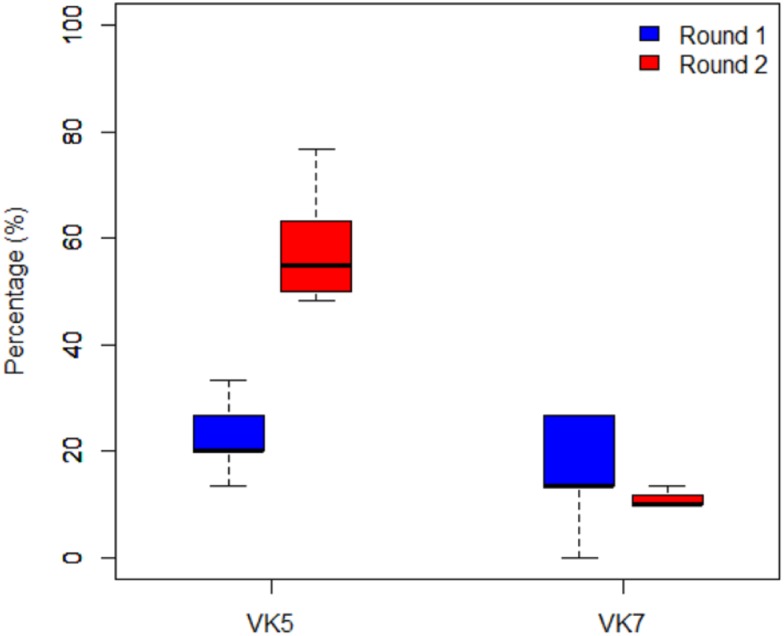
Proportions of young males in the villages of VK5 and VK7 pre-intervention (Round 1 in blue) and post-intervention (Round 2 in Red).

The proportions of Mann-Whitney young males found in VK5 more than doubled before and after intervention (W = 0; P = 0.0008) with an average of 22.92% in Round 1 and 57.3% in Round 2. However, in VK7 the proportion of young males remained low and did not significantly differ prior to an after the intervention, with 16.67% in Round 1 and 15% in Round 2 (W = 36.5; P = 0.64).

## Discussion

The ultimate goal of this study was to assess the feasibility of crashing mosquito population by targeting swarms. The study was conducted in the rice field area of Bama, Vallee du Kou, where mosquito densities are extremely high, due to the availability of extensive larval habitats. We showed that killing males in mass as well as the few mate seeking females present in the swarms at the time of intervention significantly reduced mosquito population by as much as 80%. A significant decrease in female insemination rate and a significant shift in the age structure of male population, towards younger males incapable of mating, were also observed. This is the first study that demonstrates that targeting primarily males rather than females can drastically impact mosquito population. Consequently, this study constitutes an important paradigm shift from current and past malaria vector control strategies that have essentially focused on killing female mosquitoes. As of today, no serious study had been conducted based on the premise of reducing the mosquito population by targeting males [20]. The massive deployment of treated bednets and indoor residual spraying over the last decades has drastically changed the landscape of malaria [21]. A drop of over 50% in the number of cases has been reported in more than 40 African countries since 2001. Unfortunately, low levels of transmission still persist, even in places where the coverage rate of these intervention tools is above 80%. The failure of these vector control measures to completely interrupt transmission may be due to several factors, including insecticide resistance and taxonomic and behavioral heterogeneity in vector species, and/or vector populations exhibiting atypical biting and resting behaviors. Here we showed that targeting males in swarms has the potential to cut down the high reproductive rate of malaria vectors. The sudden decrease in vector density over a period of 10 days, indicates that the intervention not only suppressed male populations, hence disrupting mating, but also suggest that female population may have also been affected as early blood seeking or ovipositing females may have been exposed to the residual effect of the airborne insecticide which can stay in the air for 15–20 minutes after the spray. The rarity of males might have also caused unmated females to spend several nights seeking swarms, thereby increasing their exposure to swarm spraying. That the intervention has clearly affected male population is without any doubt as female insemination rate significantly decreased and the age structure of the male population strongly shifted towards immature males.

These results clearly indicate that targeting swarms to deplete mosquito populations theoretically offers an unrivalled opportunity to drastically reduce mosquito-borne pathogen transmission, specifically in places where residual malaria transmission persists despite high coverage by current intervention tools. However, while the approach has proven effective against *An*. *coluzzii* in this study, it needs to be validated for other vector species. It is worth mentioning that this methodology can be used against all major malaria vectors in Africa, as they all mate in swarms [16,22,23,24,25,26,27,28,29,30,31]. Interestingly, male mosquitoes predictably use the same sites to swarm time over time, and the same locations are even used for swarming over several years [16]. Furthermore, the tight clustering of the targeted individuals in swarms provides opportunities for controlling the vectors with reduced dose space-spray applications.

Further studies need to be done to evaluate the cost-effectiveness of this intervention but also to better define the optimal spraying pattern and frequency. For example, the current study has not measured the time lap required for the population to rebound after it was crashed by swarm killing. It will also be interesting to replicate this study in different ecological settings and with different vector species. It is noteworthy that the current study was conducted at the peak of mosquito density in an ecological setting where mosquito densities were very high and that, despite this, a drastic impact on vector population was still observed. Most ecological settings in Africa have fewer mosquitoes that result in fewer swarms of smaller sizes, hence the approach is expected to have a bigger impact at less effort. Furthermore, targeting the population in the beginning of the rainy season where mosquito density is low, may deflect the growth curve of the population preventing them from reaching the usual peak densities.

## Supporting information

S1 File Data_Base. (XLSX)
